# Inflammatory stress potentiates emodin-induced liver injury in rats

**DOI:** 10.3389/fphar.2015.00233

**Published:** 2015-10-23

**Authors:** Can Tu, Dan Gao, Xiao-Fei Li, Chun-Yu Li, Rui-Sheng Li, Yan-Ling Zhao, Na Li, Ge-Liu-Chang Jia, Jing-Yao Pang, He-Rong Cui, Zhi-Jie Ma, Xiao-He Xiao, Jia-Bo Wang

**Affiliations:** ^1^China Military Institute of Chinese Medicine, 302 Military Hospital, Beijing, China; ^2^Institute of Medicinal Plant Development, Chinese Academy of Medical Sciences, Beijing, China; ^3^School of Pharmacy, Shandong University of Traditional Chinese Medicine, Jinan, China; ^4^School of Pharmacy, Chengdu University of Traditional Chinese Medicine, Chengdu, China; ^5^Department of Traditional Chinese Medicine, Beijing Friendship Hospital of Capital Medical University, Beijing, China

**Keywords:** emodin, lipopolysaccharide, idiosyncratic drug-induced liver injury, hepatotoxicity, proinflammatory mediators, therapeutic dosages

## Abstract

Herbal medicines containing emodin, widely used for the treatment of hepatitis in clinic, have been reported with hepatotoxicity in individuals. A modest inflammatory stress potentiating liver injury has been linked to the idiosyncratic drug-induced liver injury (IDILI). In this study, we investigated the hypothesis that lipopolysaccharide (LPS) interacts with emodin could synergize to cause liver injury in rats. Emodin (ranging from 20, 40, to 80 mg/kg), which is in the range of liver protection, was administered to rats, before LPS (2.8 mg/kg) or saline vehicle treatment. The biochemical tests showed that non-toxic dosage of LPS coupled with emodin caused significant increases of plasma ALT and AST activities as compared to emodin alone treated groups (*P* < 0.05). In addition, with LPS or emodin alone could not induce any changes in ALT and AST activity, as compared with the control group (0.5% CMC-Na treatment). Meanwhile, the plasma proinflammatory cytokines, TNF-α, IL-1β, and IL-6 increased significantly in the emodin/LPS groups compared to either emodin groups or the LPS (*P* < 0.05). Histological analysis showed that liver damage was only found in emodin/LPS cotreatmented rat livers samples. These results indicate that non-toxic dosage of LPS potentiates the hepatotoxicity of emodin. This discovery raises the possibility that emodin and herbal medicines containing it may induce liver injury in the inflammatory stress even in their therapeutic dosages.

## Introduction

Liver injury is a fundamental pathological process in most hepatic diseases. Chronic liver injury leads to hepatic fibrosis, liver cirrhosis, and even liver cancer (Beyoğlu and Idle, 2013; Huang et al., 2013). Accordingly, many studies have been focused on the protective effects of natural compounds or plant extracts upon various liver injury *in vivo* (Harnack et al., 2001; Bent and Ko, 2004). As penetration and usage increase, consecutive reports of hepatotoxic effects have appeared gradually, such as Shou-Wu Pian, Capsule Shengjing, and some herbs containing anthraquinones including *Polygonum multiflorum*, *Sennae fructus angustifoliae*, and *Rheum palmatum* L. (Panis et al., 2005; Stickel et al., 2005; Wang et al., 2011; Ma et al., 2015).

Emodin (1,3,8-trihydroxy-6-methyl anthraquinone) is an anthraquinone derivative from Chinese herbs *Rheum palmatum* (rhubarb), *Polygonum multiflorum*, *Cassia obtusifolia*, etc. The hepatoprotective effects of emodin have been reported in a series of studies using animal models (Zhang, 2006; Dong et al., 2009; Bhadauria, 2010), and some Chinese medicine compound prescriptions mainly containing rhubarb are often used for the treatment of hepatitis in clinic (Liang et al., 2006; Wang et al., 2010). However, recently, some studies have reported a contradictory event that emodin has hepatotoxicity to normal rats (National Toxicology Program, 2001; Wang et al., 2009). Whereas the mechanism of emodin-induced liver injuries remains to be elucidated and the risk factors of hepatotoxicity are not well-defined, a research to investigate the paradoxical effects of hepatoprotection and hepatotoxicity of emodin is necessary.

Lipopolysaccharide (LPS) is the major component of the outer membrane of Gram-negative bacteria. LPS, as a potent inflammagen, could stimulate the immune system, which subsequently releases numerous proinflammatory mediators including cytokines [e.g., tumor necrosis factor-alpha (TNF-α), interleukin-1β (IL-1β), interleukin-6 (IL-6), and other chemokines], lipid metabolites, reactive oxygen species (ROS), etc. In the past years, considerable experiments support that human idiosyncratic drug-induced liver injury (IDILI) can be reproduced in animals by concurrent exposure to low dose of LPS, which induces a modest inflammatory stress (Ganey et al., 2004; Roth and Ganey, 2011). Recent evidences from animal models indicate that a mild inflammatory microenvironment, caused by drug (such as trovafloxacin or chlorpromazine) treatment, may enhances the susceptibility to hepatotoxic damage and thereby result in a toxic effect (i.e., an “idiosyncratic” response; Barton et al., 2001; Waring et al., 2006; Deng et al., 2009). These studies suggest that emodin-induced liver injury in rats would synergize to cause hepatotoxicity upon co-treatment with a non-toxic but modestly inflammatory dose of LPS, namely, idiosyncratic emodin-induced liver injury.

In this study, LPS-stimulated rats were used as a tool to test this hypothesis by investigating the paradoxical effects of hepatoprotective or hepatotoxic activity of emodin at the dosage of hepatoprotection. Our data will not only offer significant reference for the study of susceptible hepatotoxicity and rational to use of emodin, but provide novel insight into the mechanisms of emodin in liver protection.

## Materials and Methods

### Reagents

Lipopolysaccharide derived from Escherichia coli serotype 055:B5 was used (Sigma-Aldrich, Inc., USA). Emodin and sodium pentobarbital were purchased from the Sigma-Aldrich (St. Louis, USA). Carboxymethylcellulose sodium (CMC-Na) and formaldehyde were supplied by the Xilong Chemical Co., Ltd. (Guangdong, China). The disposable vacuum blood vessels and collection needles were purchased from the Tianjin Hanaco Medical Materials Co., Ltd. (Tianjin, China). Alanine transaminase (ALT), aspartate aminotransferase (AST) activity and total bile acid (TBA) kits were purchased from the Jiancheng bioengineering institute (Nanjing, China). Tumor necrosis factor-α (TNF-α), interleukin-1β (IL-1β) and interleukin-6 (IL-6) ELISA kits were purchased from the Dakewe for Biological Technology Co., Ltd. (Beijing, China).

### Animals and Treatments

All studies *in vivo* were conducted in the Laboratory Animal Center of the 302 Military Hospital. Experimental rats are maintained in accordance with the National Institutes of Health Guide for the Care and Use of Laboratory Animals, and experimental procedures were approved by the Committee on the Ethics of Animal Experiments of the 302 Military Hospital (Approval ID: 11- 037). Male Sprague-Dawley (SD) rats weighing 180 to 200 g were used for these studies. Animals were allowed to standard chow and water *ad libitum* under standard husbandry conditions (22 + 2°C temperature, 60–80% relative humidity and 12 h photoperiod). They were allowed to acclimate for 1 week in a 12 h light/dark cycle with lights turned on at 9:00 am.

The experimental animals are clustered into 4 groups: control group (0.5% CMC-Na treatment), LPS group (2.8 mg/kg), emodin groups (20, 40, and 80 mg/kg, respectively), LPS/emodin groups (2.8/20 mg/kg, 2.8/40 mg/kg, and 2.8/80 mg/kg, respectively). The doses of LPS and emodin were used in a condition that drug treatment did not cause a significant injury for rats. Rats were fasted for 12 h only with water *ad libitum*, then received intragastric administration of 0.5% CMC-Na mixture only as control and 20, 40, and 80 mg/kg emodin (dissolved in 0.5% CMC-Na) for groups of emodin, LPS/emodin treatments. 3 h later, rats were given 2.8 mg/kg LPS solution for experimental groups (LPS, LPS/emodin) by tail injection. 7 h later, rats were anesthetized with sodium pentobarbital (70 mg/kg i.p.), and plasma were collected with a syringe containing sodium citrate by drawing blood from the vena cava, then euthanized. Plasma was stored at –20°C for further analysis and representative slices (1 cm) of the ventral portion of the left lateral liver lobe was fixed in 10% neutral buffered formalin for subsequent liver biochemical tests and histological assessment of hepatic pathology.

### Plasma Indicators and Histopathology Assessment

Hepatic parenchymal cell injury was estimated by evaluating plasma ALT, AST activity and TBA content using the Hitachi clinical analyzer 7020 (Hitachi High-Technologies Co., Japan). Inflammatory microenvironment was assessed by measuring plasma TNF-α, IL-1β, and IL-6 contents using ELISA kits according to the manufacturer’s protocol. Formalin fixed liver samples were routinely processed and embedded in paraffin, sectioned to a thickness of approximately 5 μm, and stained with hematoxylin and eosin (HE).

### Statistical Analysis

All the data presented in the manuscripts were analyzed using the Statistical Package for the Social Sciences of Windows, version 17.0 (SPSS Inc., Chicago, IL, USA). The inter-group variation was measured by one-way analysis of variance (ANOVA). Difference was considered statistically significant when *P* ≤ 0.05, and very significant when *P* ≤ 0.01. The error bars represents the “SD” value (standard deviation).

## Results

### Common Changes in Rats

After observing and recording the external manifestations of rats, they were administered by intragastric administration of emodin or tail intravenously LPS. In control group, rats with smooth body hair, urine, and spirit are normal as before. In emodin only groups, rats with loose stools, soft stool, messy body hair, yellow urine are appeared. In LPS/emodin groups, the activity of rats are becoming significantly slow, sluggish, listlessness, except for loose stools, messy body hair, and yellow urine, especially in the LPS/emodin (2.8/80 mg/kg), whose phenomenon was more obvious even with animal death.

### Plasma Biochemical Index

**FIGURE 1:**
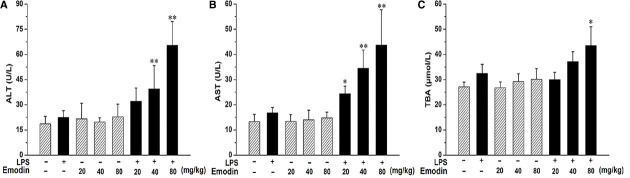
**The Plasma biochemical indicators in the absence and presence of LPS with emodin.** Rats were treated with various doses of emodin (20, 40, or 80 mg/kg, i.g.) or its vehicle (0.5% CMC-Na). After 3 h, LPS or emodin/LPS groups animals received LPS (2.8 mg/kg, i.v.) or its vehicle (0.5% CMC-Na). **(A)** and **(B)** Blood samples were taken at 7 h after the administration of LPS, and plasma ALT and AST levels were measured. **(C)** Plasma TBA content were measured from the same conditions. The results were presented as mean ± SD from of 10 rats. Significance of differences was from the value of emodin/LPS rats (*P < 0.05, **P < 0.01 vs emodin groups). For groups, “+” and “–” represent “presence” and “absence,” respectively.

Treatment of rats with emodin or LPS alone did not cause an increase in plasma ALT, AST activity and TBA content, (Figure 1). However, rats had significant increase in plasma ALT and AST activities after co-treatment with 20, 40, or 80 mg/kg emodin plus LPS as well as in TBA content with 80 mg/kg emodin plus LPS, compared with emodin groups. The phenomenon indicates that emodin or LPS alone did not induce liver injury in rats at any of the doses given, while rats had significant liver injury after co-treatment.

### Plasma Proinflammatory Cytokine Assessment

**FIGURE 2:**
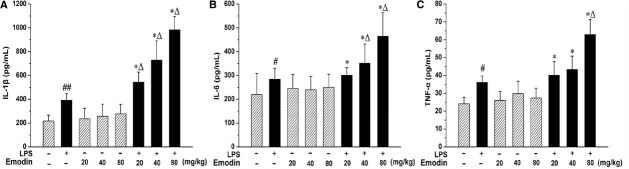
**Chemokine and cytokine expression in rats cotreated with LPS and emodin.** Rats were treated with emodin (20, 40, or 80 mg/kg, i.g.) or its vehicle (0.5% CMC-Na) and LPS (2.8 mg/kg, i.v.) or its vehicle (0.5% CMC-Na) as described in Figure 1. (A–C) Showed Plasma proinflammatory cytokine levels determined by ELISA for IL-1β, IL-6, and TNF-α respectively. The data were presented as mean ± SD of 10 rats, and statistically significant difference (*P < 0.05, vs emodin groups; Δ*P* < 0.05, vs LPS group; #*P* < 0.05 and ##*P* < 0.01, vs control).

**FIGURE 3:**
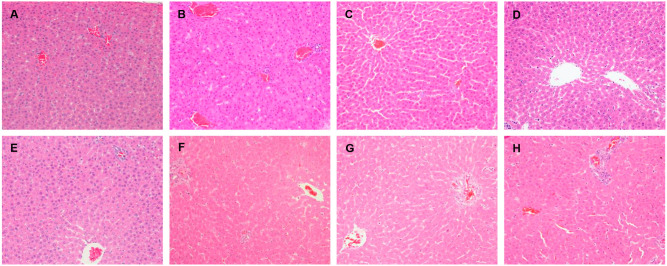
**Histopathological damage in rats liver given LPS, emodin only and cotreated with LPS/emodin. (A)** Liver sections from rats were treated with vehicle (0.5% CMC-Na); **(B)** with LPS (2.8 mg/kg); **(C)** emodin (20 mg/kg); **(D)** emodin (40 mg/kg); **(E)** emodin (80 mg/kg); **(F)** LPS/emodin (20 mg/kg); **(G)** LPS/emodin (40 mg/kg); **(H)** LPS/emodin (80 mg/kg). Liver samples were collected at 7 h after LPS tail intravenously injection (HE stained, × 200 magnification) and HE staining was performed to investigate the histological changes in all experimental groups.

Lipopolysaccharide challenge caused marked increases in the levels of the proinflammatory cytokines TNF-α, IL-1β, and IL-6 (*P* < 0.01 or *P* < 0.05) when compared with saline-treated controls (Figure 2). Although trace amounts of LPS could cause mild inflammation in rats, the HE staining from the liver sections showed that those LPS alone injected rats do not develop obvious hepatocellular injury (Figure 3). Furthermore, the plasma proinflammatory cytokines levels are significantly higher in the emodin/LPS groups comparing to either emodin groups or LPS group (*P* < 0.05). The results demonstrated that non-toxic dosage of LPS potentiated the hepatic inflammation in the hepatoprotective doses of emodin.

### Liver Histopathology Assessment

Shown in Figure 3, no significantly histologic lesions were observed in both control and emodin groups, since no obvious histological lesion emerged. The hepatic lobule was clear, liver cells were uniform, the nucleus was clearly visible, and liver cells surrounding the central veins were distributed in radial arrangement of liver cell cords. LPS-treated groups showed evidences of mild pathological alteration: slight inflammatory cells invasion in portal area around the blood vessels, a small amount of kupffer cell proliferation and enlargement, which showed inflammatory signaling pathways were activated. However, there were significantly pathological changes in LPS/emodin groups: hepatic blood sinus congestion expanded obviously near the central vein, part of the liver cell visible swelling, some cells have no nuclei even dot necrosis, and a large number of inflammatory cells infiltration in portal area around the blood vessels. In figure H, liver cell injury increased significantly and randomly distributed, as well as local necrosis of liver cells existed, which was not observed in any of the livers from rats treated with LPS or emodin alone.

## Discussion

In current studies, in order to investigate the hepatotoxicity induced by the anthraquinone derivative emodin, we applied a rat model of drug-inflammation interaction. Our data indicates that emodin or LPS alone did not increase the clinical chemical biomarkers of liver injury or cause any lesions in the liver. However, in emodin/LPS-co-treated rats, serum markers of hepatocellular injury were increased significantly, and foci of necrotic parenchymal cells were found in livers. In clinic, Chinese patent medicine containing emodin are proved with a hepatoprotection role (Fang et al., 2011). Previous studies have shown that animals treated with clinical equivalent dose of emodin do not develop liver injury, while high dose and long term treatment could induce hepatotoxicity (Zeng et al., 2013). In Table 1, the data revealed that the hepatotoxic dose of emodin was much higher than the hepatoprotective dose of that in animals (Zhan et al., 2000; Dang et al., 2008; Lei et al., 2008; Wang et al., 2009; Zhao et al., 2009). Wang et al. (2009) treated rats for 3 weeks with rhubarb with a dose of 3 g/kg (equivalent to emodin with the dose of 45 mg/kg) and observed liver injuries. It is highly possible that accumulative dose (around 945 mg/kg) could be danger to the rat liver cells since livers are the major organs for the drug metabolism. However, in our experiment, emodin coupled with LPS could induce liver injury even in their therapeutic doses (20, 40, and 80 mg/kg), accompanied by the immune cell infiltration to the livers. This data is interesting since LPS-mediated immune system activation promotes the liver injury. In this way, the hepatotoxic effect of emodin treatment maybe a hidden danger to patients with IDILI. Potentially, our research helps to distinguish drugs, which is in the same pharmacologic class but has idiosyncratic toxicity, to avoid adverse drug reactions greatly.

**TABLE 1:** **The hepatoprotection and hepatotoxicity of emodin in animals**.

		**Administration**			
**Drug**	**Species**	**Dosage**	**Period**	**Effect**	**Reference**
Emodin	Rat	20 mg/kg	12 week	Protective	Dong et al., 2009
Emodin	Rat	40 mg/kg	36 h	Protective	Zhao et al., 2009
Emodin	Rat	40 mg/kg	12 week	Protective	Dang et al., 2008
Emodin	Rat	20,40, 80 mg/kg	6 week	Protective	Zhan et al., 2000
Emodin	Rat	406 mg/kg	1 times	Toxic	Lei et al., 2008
	Mouse	580 mg/kg			
Emodin	Rat	340 mg/kg	14 week	Toxic	National Toxicology Program, 2001
	Mouse	800 mg/kg			
Rhubarb	Rat	^a^38, 76 g/kg	4 week	Toxic	Ma, 2007
Rhubarb	Rat	^b^14.7, 40 g/kg	4 week	Toxic	Wang et al., 2010
Rhubarb	Rat	^c^20 g/kg	3 week	Toxic	Wang et al., 2009

The total content of emodin generally presents 1.5∼3% in rhubarb and was calculated according to its content of 1.5%; ^a^38 and 76 g/kg Rhubarb were amounted to 570 and 1140 mg/kg of emodin; ^b^14.7 and 40 g/kg Rhubarb is the equal of 220 and 600 mg emodin; ^c^20 g/kg Rhubarb equals 300 mg/kg emodin.

Idiosyncratic adverse drug response is a type of adverse reaction that occurs in a minority of patients during drug therapy. Liver is one of the major organ targets (Roth and Ganey, 2011). Emodin with hepatic protective effect, is reported of hepatotoxic effects in some cases. We suspected that it might be associated with hepatic idiosyncratic adverse drug reactions (IADRs), due to no significant correlation between age, gender, dose and adverse reactions of some herbs containing it in clinic. However, the mechanism of emodin-induced liver injury has not been clarified because of the lack of experimental animal models. Previous study has been proposed that inflammatory stress may render an individual susceptible to IADRs, and numerous IDILI have been developed in LPS/drug models that support the inflammatory stress hypothesis. LPS binds to Toll-like receptors (TLRs), which is primary signaling receptors for LPS, initiates immune activating signals and leads to stimulation of inflammatory cells and consequent expression and release of numerous proinflammatory mediators (Testro and Visvanathan, 2009; Guo and Friedman, 2010). These mediators, including cytokines (TNF-α, IL-1β, IL-6, etc.), toxic proteases, ROS, might increase liver cell sensitivity to drugs when the response is modest or lead to liver injury in aggravated conditions (Gandhi et al., 2013). In addition, clinical chemistry (especially liver enzymes ALT and AST) and histopathology data are hallmark indicators of hepatotoxicity in animal models (Zou, 2010). We evaluated the utility of LPS/drug models in terms of cytokines, clinical chemistry and histopathology to explore mechanisms of emodin-induced liver injury. When given alone, none of the doses of emodin tested (20–80 mg/kg) produced significant increases in serum cytokines such as TNF-α, IL-1β and IL-6. However, when coadministered with non-injurious LPS which induces modest inflammation, cytokine levels are significantly enhanced at the emodin dose of 20 mg/kg and greater. Neither emodin nor LPS given alone had a significant hepatotoxic effect as measured by ALT and AST activity compared to control animals. In contrast, co-treatment of rats with emodin/LPS led to a significant increase in liver injury markers expressions (ALT, AST, and TBA). The results might indicate proinflammatory episode increased liver cell sensitivity, which transformed therapeutic dosages of emodin into toxic doses and led to liver injury. Other drugs associated with idiosyncratic hepatotoxicity in humans, such as ranitidine and trovafloxacin, also had a synergistic effect on the LPS-mediated models similarly.

*Glycyrrhiza*, possessing several pharmacological activities including anti-inflammatory and hepatoprotective, has been used as herbal medicine worldwide for over 4000 years (Shibata, 2000; Yoshida et al., 2007). As the main active ingredient of *Glycyrrhiza*, glycyrrhizin is reported to suppress the LPS sensor toll-like receptor 4 (TLR4)/Myeloid differentiation protein 2 (MD-2) complex signaling, result in attenuating LPS-mediated inflammatory response (Kim et al., 2008; Schrofelbauer et al., 2009; Honda et al., 2012). According to traditional Chinese Medicine (TCM) theories, compatibility of medicines are usually used to reach the goal of better curative efficacies and fewer side effects, just like Rhubarb and *Glycyrrhiza* were used together in one prescription (Cao and Wang, 2013). Typically, “Rhubarb *Glycyrrhiza* Soup,” from the ancient medical book Synopsis of Golden Chamber written by the medical sage Zhang Zhongjing, is a classic clinical decoction, which has powerful curative effects in treating diabetes, severe acute pancreatitis, detoxification, liver and renal injury, and is widely used till now (Han et al., 2010). Most TCM practitioners in modern times commonly believe that *Glycyrrhiza* may harmonize and modify Rhubarb (Shen et al., 2013). We predict that glycyrrhizin of *Glycyrrhiza* can regulate TLR4 signaling path, decrease production of proinflammatory cytokines to alleviate the idiosyncratic liver injury of emodin absorbed into bloodstream, which maybe a most likely hypothesis giving a new approach for future researches.

## Conclusion

In summary, inflammation response could decrease the threshold for toxicity, making an individual more susceptible to some idiosyncratic hepatotoxicants. In this study, a modest inflammatory episode induced by non-injurious LPS as the idiosyncratic liver injury model potentiates emodin-induced liver injury in rats, whereas neither LPS nor emodin was hepatotoxic alone. Although the mechanisms underlying these are not yet fully understood, the finding raise the possibility that patients experiencing an inflammatory response who concurrently consume alternative medicines containing emodin maybe susceptible to liver injury.

## Author Contributions

CT, DG, and X-fL performed the experiments, analyzed the data and wrote the manuscript. C-yL, R-sL, and Y-lZ collected and prepared samples. NL, G-l-cJ, and J-yP performed the analyses. H-r C and Z-j M amended the paper. J-bW and X-hX designed the study and amended the paper.

### Conflict of Interest Statement

The authors declare that the research was conducted in the absence of any commercial or financial relationships that could be construed as a potential conflict of interest.
