# Corrigendum: Inflammatory Stress Potentiates Emodin-Induced Liver Injury in Rats

**DOI:** 10.3389/fphar.2020.597772

**Published:** 2021-01-25

**Authors:** Can Tu, Dan Gao, Xiao-Fei Li, Chun-Yu Li, Rui-Sheng Li, Yan-Ling Zhao, Na Li, Ge-Liu-Chang Jia, Jing-Yao Pang, He-Rong Cui, Zhi-Jie Ma, Xiao-He Xiao, Jia-Bo Wang

**Affiliations:** ^1^ China Military Institute of Chinese Medicine, 302 Military Hospital, Beijing, China; ^2^ Institute of Medicinal Plant Development, Chinese Academy of Medical Sciences, Beijing, China; ^3^ School of Pharmacy, Shandong University of Traditional Chinese Medicine, Jinan, China; ^4^ School of Pharmacy, Chengdu University of Traditional Chinese Medicine, Chengdu, China; ^5^ Department of Traditional Chinese Medicine, Beijing Friendship Hospital of Capital Medical University, Beijing, China

**Keywords:** emodin, lipopolysaccharide, hepatotoxicity, therapeutic dosages, proinflammatory mediators, idiosyncratic drug-induced liver injury

In the original article, there was a mistake in Figure 3D as published. During the histological evaluation, the histological images were photographed by microscope in different fields of each animal and named as the number of animal and groups, which were placed under the same folder of group name. Because of our unpremeditated mistake, the last photo of microscopic field of the animal in group C (emodin, 20 mg/kg) was wrongly named as group D (emodin, 40 mg/kg) and then saved in the group D folder. This unpremeditated mistake caused a coincidental error of picking out the typical photo of each group in preparing Figure 3, that a wrongly named group C photo was picked out from group D folder and was wrongly regarded as group D photo. This does not impact on the interpretation of the data results. In groups C and D, 20 or 40 mg/kg of emodin were administered respectively to normal rats, which showed no significantly histological lesions in either group C or group D. All the animals in the two groups showed accordant results of no histologic lesions and the serum hepatic biochemistry indices did not reveal alterations. These results are consistent with the literatures and expectable as the dosages of emodin were significantly lower than the toxic dosage of this compound in normal rats. Thus, this corrigendum affects neither the interpretation of the data nor conclusions of this work. The corrected Figure 3 appears below.

**FIGURE 3:**
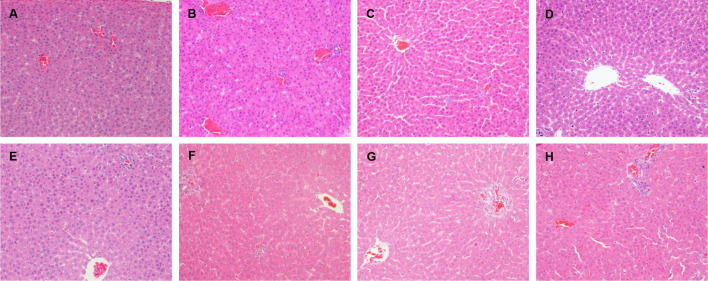
Histopathological damage in rats liver given LPS, emodin only and cotreated with LPS/emodin. **(A)** Liver sections from rats were treated with vehicle (0.5% CMC-Na); **(B)** with LPS (2.8 mg/kg); **(C)** emodin (20 mg/kg); **(D)** emodin (40 mg/kg); **(E)** emodin (80 mg/kg); **(F)** LPS/emodin (20 mg/kg); **(G)** LPS/emodin (40 mg/kg); **(H)** LPS/emodin (80 mg/kg). Liver samples were collected at 7 h after LPS tail intravenously injection (HE stained, ×200 magnification) and HE staining was performed to investigate the histological changes in all experimental groups.

The authors apologize for this error and state that this does not change the scientific conclusions of the article in any way. The original article has been updated.

